# Supplementary material for: Microclimate factors related to dengue virus burden clusters in two endemic towns of Mexico
Source: PLoS One. 2024 Jun 6;19(6):e0302025. doi: 10.1371/journal.pone.0302025 (PMC11156286; doi:10.1371/journal.pone.0302025)
Supplement: S1 Fig — (PDF) [file pone.0302025.s001.pdf]

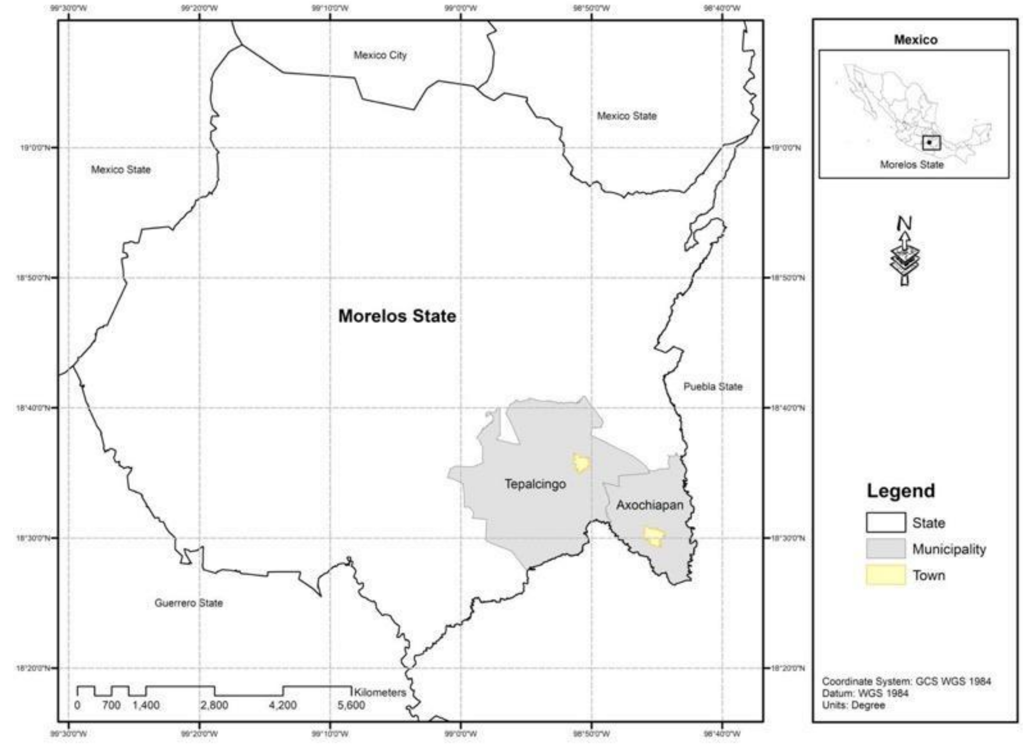

S1 Figure. Geographical location of the localities of Axochiapan and Tepalcingo - State of Morelos, Mexico.
