# Supplementary figures and images for: Microclimate factors related to dengue virus burden clusters in two endemic towns of Mexico
Source: PLoS One. 2024 Jun 6;19(6):e0302025. doi: 10.1371/journal.pone.0302025 (PMC11156286; doi:10.1371/journal.pone.0302025)

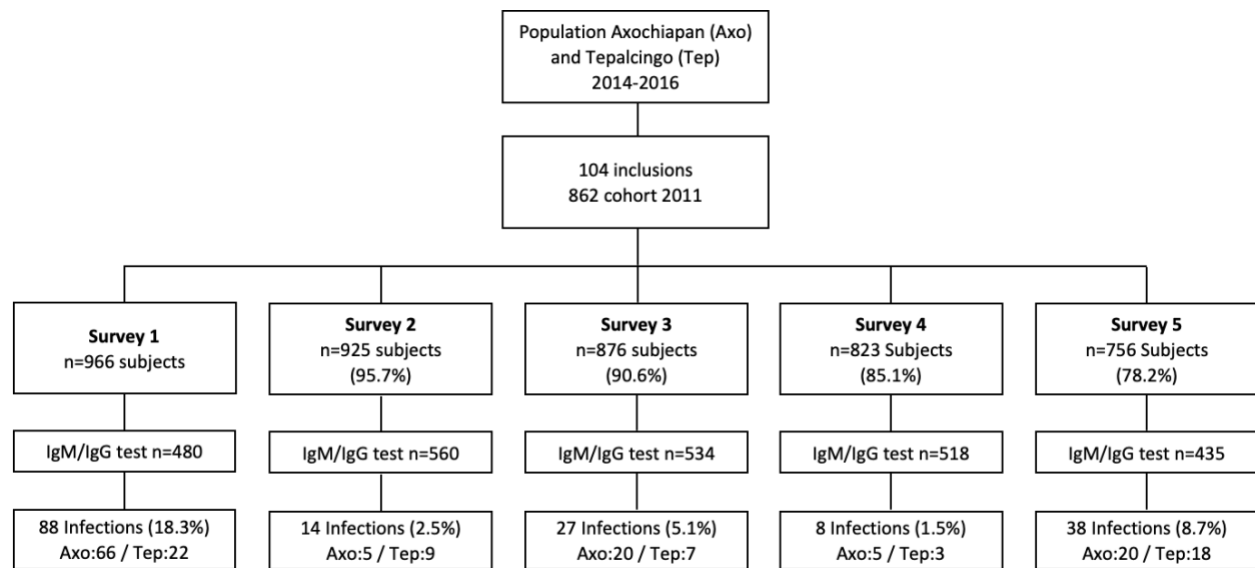

**S2 Figure. Description of follow-up and recent DENV infection.**

Supplement: S2 Fig — (PDF) [file pone.0302025.s002.pdf]
