# Supplementary material for: Microclimate factors related to dengue virus burden clusters in two endemic towns of Mexico
Source: PLoS One. 2024 Jun 6;19(6):e0302025. doi: 10.1371/journal.pone.0302025 (PMC11156286; doi:10.1371/journal.pone.0302025)
Supplement: S9 Fig — A. Spatial distribution of the participants (Red dots: Positive for recent DENV infection; Green dots: Negative for recent DENV infection). B. Percentage of recent DENV infection. C. Hot Spots of recent DENV infection. D. Clusters (Autocorrelation, Anselin local Moran’s I). Sources: Esri module of ArcGIS, DigitalGlobe, GeoEye, Earthstar Geographics, CNES/Airbus DS, USDA, USGS, AeroGRID, IGN, and the GIS User Community. (PDF) [file pone.0302025.s009.pdf]

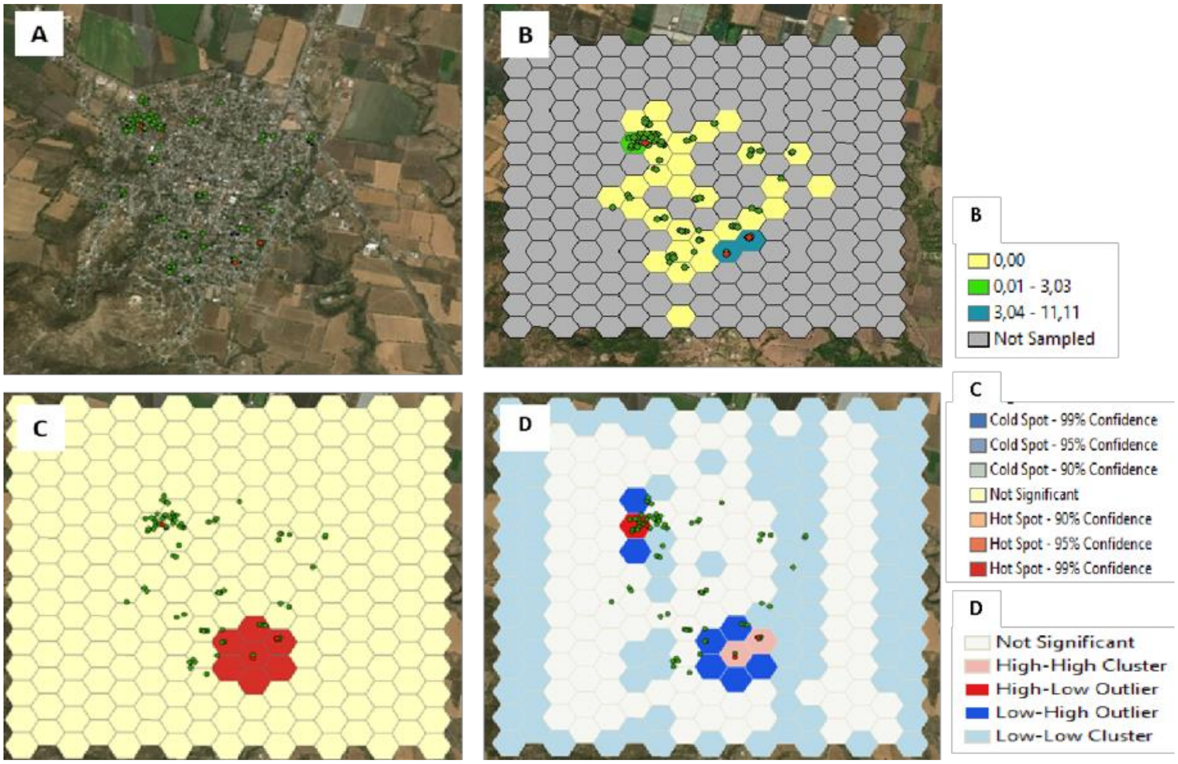

S9 Figure. Spatial distribution and clusters of recent DENV infections in Tepalcingo, fourth survey.
A. Spatial distribution of the participants (Red dots: Positive for recent DENV infection; Green dots: Negative for recent DENV infection). B. Percentage of recent DENV infection. C. Hot Spots of recent DENV infection. D. Clusters (Autocorrelation, Anselin local Moran's I). Sources: Esri module of ArcGIS, DigitalGlobe, GeoEye, Earthstar Geographics, CNES/Airbus DS, USDA, USGS, AeroGRID, IGN, and the GIS User Community.
