# Supplementary material for: Microclimate factors related to dengue virus burden clusters in two endemic towns of Mexico
Source: PLoS One. 2024 Jun 6;19(6):e0302025. doi: 10.1371/journal.pone.0302025 (PMC11156286; doi:10.1371/journal.pone.0302025)
Supplement: S13 Fig — Red dots: Positive for recent DENV infection; Green dots: Negative for recent DENV infection. A. Survey 1. B. Survey 2. C. Survey 3. D. Survey 4. E. Survey 5. Sources: Esri module of ArcGIS, DigitalGlobe, GeoEye, Earthstar Geographics, CNES/Airbus DS, USDA, USGS, AeroGRID, IGN, and the GIS User Community. (PDF) [file pone.0302025.s013.pdf]

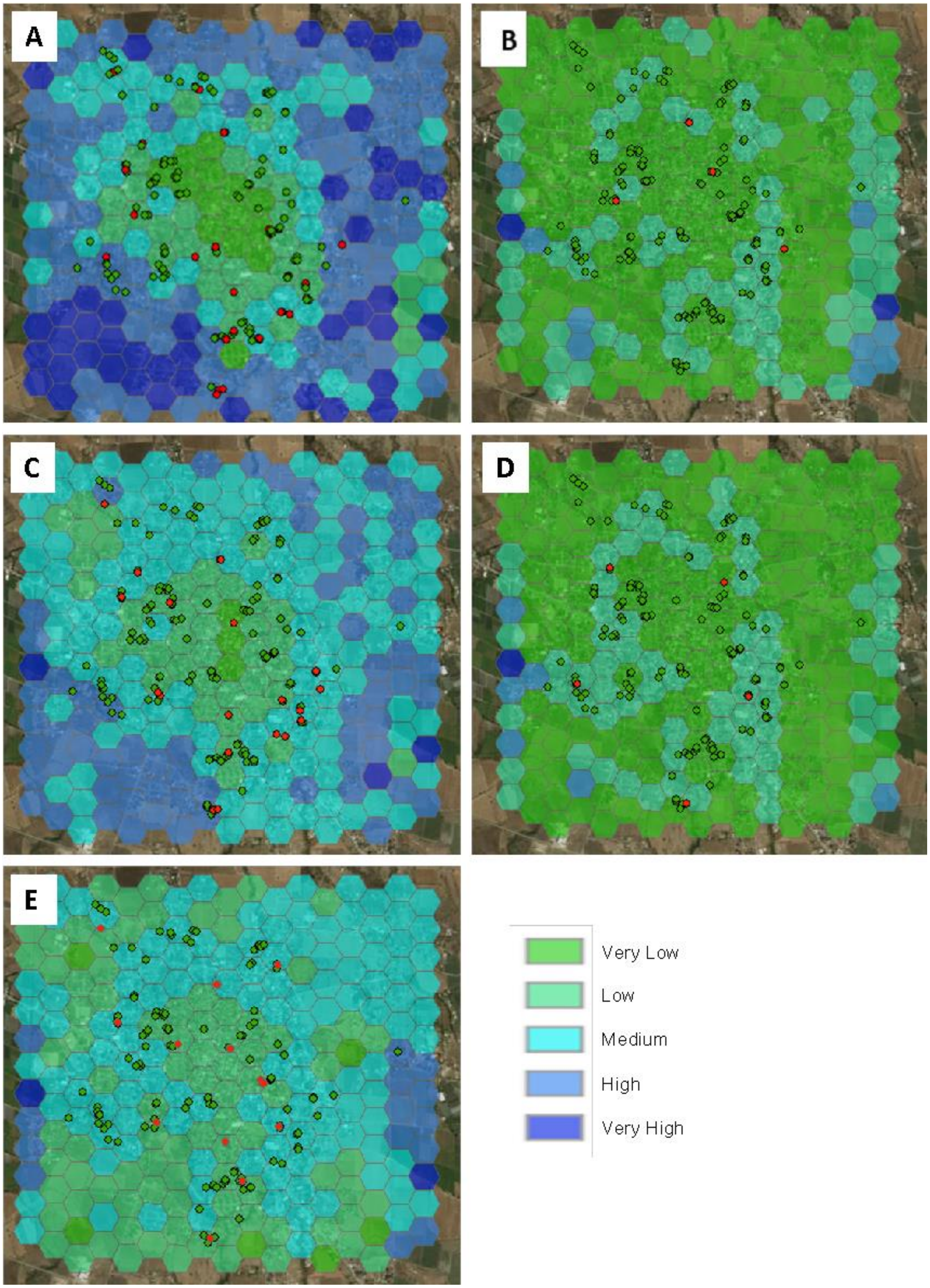

S13 Figure. Spatial distribution of soil humidity in Axochiapan by survey.
Red dots: Positive for recent DENV infection; Green dots: Negative for recent DENV infection. A. Survey 1. B. Survey 2. C. Survey 3. D. Survey 4. E. Survey 5. Sources: Esri module of ArcGIS, DigitalGlobe, GeoEye, Earthstar Geographics, CNES/Airbus DS, USDA, USGS, AeroGRID, IGN, and the GIS User Community.
