# Supplementary material for: Microclimate factors related to dengue virus burden clusters in two endemic towns of Mexico
Source: PLoS One. 2024 Jun 6;19(6):e0302025. doi: 10.1371/journal.pone.0302025 (PMC11156286; doi:10.1371/journal.pone.0302025)
Supplement: S1 Table — (PDF) [file pone.0302025.s017.pdf]

S1 Table. Sociodemographic characteristics of the study population by survey.

	**Survey 1**	**Survey 2**	**Survey 3**	**Survey 4**	**Survey 5**
**Characteristic**	n=480	n=560	n=534	n=518	n=435
**Age** years median (IQR)	22 (13 - 38)	24 (14 - 41)	24 (14 - 41)	24 (14 - 41)	24 (13 - 41)
**Sex** Male n (%)	206 (42.9)	230 (41.1)	218 (40.8)	209 (40.3)	171 (39)
**Locality** Axochiapan n (%)	324 (67.5)	381 (68)	359 (67.1)	348 (67.2)	284 (64.9)
**Occupation n (%)**					
**Student**	178 (37.1)	187 (33.4)	179 (33.5)	175 (33.8)	157(35.8)
Dependent employee	96 (20)	117 (20.9)	111 (20.7)	107 (20.7)	85 (19.4)
Self-employed	61 (12.7)	75 (13.4)	74 (13.8)	66 (12.7)	54 (12.3)
Housewife	119 (24.8)	150 (26.8)	143 (26.7)	141 (27.2)	121 (27.6)
Unemployed	25 (5.2)	30 (5.4)	27 (5)	28 (5.4)	20 (4.6)
Other	1 (0.2)	1 (0.2)	1 (0.2)	1 (0.2)	1 (0.2)
**Educational Level** n (%)					
Illiterate	49 (10.2)	60 (10.7)	55 (10.3)	52 (10)	47 (10.7)
Read or write	70 (14.6)	81 (14.5)	77 (14.4)	73 (14.1)	65 (14.8)
Basic	105 (21.9)	116 (20.7)	112 (21)	110 (21.2)	97 (22.2)
Secondary	151 (31.5)	174 (31.1)	167 (31.2)	160 (30.9)	138 (31.5)
Preparatory	70 (14.5)	83 (14.8)	81 (15.1)	77 (14.8)	59 (13.5)
Higher education	35 (7.3)	46 (8.2)	43 (8.0)	46 (8.8)	32 (7.31)
**Years of study completed** median (IQR)	8 (5 - 10)	9 (5 - 11)	9 (5 - 11)	9 (5 - 11)	8 (5 - 10)
**Social Security** n (%)					
Public	386 (80.6)	452 (80.8)	433 (81.1)	418 (80.1)	355 (81.2)
Uninsured	51 (10.6)	62 (11.1)	58 (10.9)	54 (10.4)	41 (9.4)
Private	42 (8.8)	45 (8)	43 (8)	45 (8.7)	41 (9.4)
**Has lived all life in Morelos (yes)** n (%)	401 (83.5)	470 (84)	456 (85.2)	443 (85.5)	379 (86.5)
**Recent DENV infection** n (%)	88 (18.3)	14 (2.5)	27 (5.1)	8 (1.5)	38 (8.7)
