# Supplementary material for: Microclimate factors related to dengue virus burden clusters in two endemic towns of Mexico
Source: PLoS One. 2024 Jun 6;19(6):e0302025. doi: 10.1371/journal.pone.0302025 (PMC11156286; doi:10.1371/journal.pone.0302025)
Supplement: S2 Table — (PDF) [file pone.0302025.s018.pdf]

S2 Table. Characteristics of the houses by survey.

Housing Characteristics	Survey 1	Survey 2	Survey 3	Survey 4	Survey 5
	n=238	n=250	n=238	n=235	n=218
**Sewer system**	n (%)	n (%)	n (%)	n (%)	n (%)
Public network	215 (90.3)	222 (88.8)	216 (90.8)	213 (90.7)	198 (90.8)
Septic tank	16 (6.7)	18 (7.2)	13 (5.5)	13 (5.5)	13 (6.0)
Ravine or crack	3 (1.3)	3 (1.2)	3 (1.3)	2 (0.9)	3 (1.4)
Not specified	4 (1.7)	7 (2.8)	6 (2.5)	7 (3.0)	4 (1.8)
**Sanitary**					
Direct discharge of water	95 (39.9)	97 (38.8)	93 (39.1)	94 (40.0)	85 (39.0)
Manual discharge of water	138 (58.0)	144 (57.6)	137 (57.7)	131 (55.7)	126 (57.8)
Without water	1 (0.4)	2 (0.8)	1 (0.4)	2 (0.9)	2 (0.9)
Other/Not specified	4 (1.7)	7 (2.8)	7 (2.9)	8 (3.4)	5 (2.3)
**Availability of piped water**					
Public network inside the house	163 (68.5)	166 (66.4)	161 (67.7)	162 (68.9)	148 (67.9)
Public network outside the house	6 (2.5)	7 (2.8)	6 (2.5)	5 (2.1)	6 (2.8)
Public tap or hydrant	2 (0.8)	2 (0.8)	2 (0.8)	2 (0.9)	2 (0.9)
Water from another house	5 (2.1)	8 (3.2)	6 (2.5)	5 (2.1)	5 (2.3)
Well Water	58 (24.4)	60 (24.0)	57 (24.0)	54 (23.0)	53 (24.3)
Other/Not specified	4 (1.7)	7 (2.8)	6 (2.5)	7 (3.0)	4 (1.8)
**Flooring material**					
Soil	5 (2.1)	6 (2.4)	5 (2.1)	4 (1.7)	4 (1.8)
Cement	227 (95.4)	236 (94.4)	225 (94.5)	222 (94.5)	208 (95.4)
Wood	2 (0.8)	1 (0.4)	1 (0.4)	1 (0.4)	1 (0.5)
Other/Not specified	4 (1.7)	7 (2.8)	7 (2.9)	8 (2.9)	5 (2.3)
**TV**	231 (97.1)	240 (96.0)	228 (95.8)	224 (95.3)	210 (96.3)
**Refrigerator**	217 (91.2)	224 (89.6)	218 (91.6)	212 (90.2)	198 (90.8)
**Washing machine**	178 (74.8)	180 (72.0)	175 (73.5)	175 (74.5)	160 (73.4)
**Computer**	77 (32.4)	76 (30.4)	71 (29.8)	73 (31.1)	64 (29.4)
**Air conditioning**	6 (2.5)	7 (2.8)	5 (2.1)	6 (2.6)	5 (2.3)
**Screens on doors**					
No	162 (68.1)	168 (67.2)	163 (68.5)	164 (69.8)	156 (71.6)
All doors	35 (14.7)	35 (14.0)	32 (13.5)	30 (12.8)	24 (11.0)
Some doors	36 (15.1)	38 (15.2)	35 (14.7)	32 (13.6)	32 (14.7)
Not specified	5 (2.1)	9 (3.6)	8 (3.4)	9 (3.8)	6 (2.8)
**Screens on windows**					
No	133 (56.6)	140 (57.3)	134 (57.7)	135 (59.2)	128 (59.8)
All windows	52 (22.1)	53 (21.7)	49 (21.1)	46 (20.1)	40 (18.7)
Some windows	48 (20.4)	48 (19.7)	47 (20.2)	45 (19.7)	44 (20.6)
Not specified	5 (2.1)	9 (3.6)	8 (3.4)	9 (3.8)	6 (2.8)
**DENV Seroprevalence** Median (IQR)	100 (75 - 100)	100 (80 - 100)	100 (80 - 100)	100 (80 - 100)	100 (100 - 100)
