# Supplementary material for: Microclimate factors related to dengue virus burden clusters in two endemic towns of Mexico
Source: PLoS One. 2024 Jun 6;19(6):e0302025. doi: 10.1371/journal.pone.0302025 (PMC11156286; doi:10.1371/journal.pone.0302025)
Supplement: S3 Table — (PDF) [file pone.0302025.s019.pdf]

S3 Table. Significant clusters of recent DENV infection in Axochiapan.

Survey	Polygon ID	% Recent DENV infections	Z-score	p value	N° Neighbors
1	60	20	2.438	0.04	4
1	122	35.3	4.070	0.008	3
1	149	100	2.893	0.024	3
1	150	40	4.482	0.022	2
1	163	10	3.475	0.026	3
1	164	25	3.911	0.016	3
3	122	5.6	4.591	0.014	3
3	149	33.3	3.044	0.038	3
3	150	33.3	4.321	0.024	2
3	163	18	6.403	0.004	3
