# Supplementary material for: Microclimate factors related to dengue virus burden clusters in two endemic towns of Mexico
Source: PLoS One. 2024 Jun 6;19(6):e0302025. doi: 10.1371/journal.pone.0302025 (PMC11156286; doi:10.1371/journal.pone.0302025)
Supplement: S4 Table — (PDF) [file pone.0302025.s020.pdf]

S4 Table. Significant Clusters of Recent DENV Infection in Tepalcingo.

Survey	Polygon ID	% Recent DENV infections	Z-score	p value	N° Neighbors
1	87	25	3.576	0.01	6
1	135	40	2.204	0.048	3
2	70	9.1	4.510	0.004	6
2	152	7.7	5.426	0.012	3
4	70	11.1	5.268	0.034	6
4	71	10	7.258	0.02	3
5	69	12.5	4.468	0.004	5
5	84	20	3.880	0.038	3
