# Supplementary material for: Microclimate factors related to dengue virus burden clusters in two endemic towns of Mexico
Source: PLoS One. 2024 Jun 6;19(6):e0302025. doi: 10.1371/journal.pone.0302025 (PMC11156286; doi:10.1371/journal.pone.0302025)
Supplement: S5 Table — *Poor housing maintenance: houses were those having a poor structure, poor organization, dirty, with unpainted or cracked walls, improvised sections, or broken windows/doors **House with larvae or pupae of Aedes. (PDF) [file pone.0302025.s021.pdf]

S5 Table. Housing characteristics related to DENV burden clusters, univariable analysis.

Housing Characteristics	n/n° Groups	PR	95% CI	*p*
**Total number inhabitants**	1132/263	1.018	0.937 - 1.106	0.671
**Inhabitants ≤5 years**	1132/263	1.042	0.796 - 1.364	0.765
**DENV % Seroprevalence**	1158/269	1.006	0.993 - 1.020	0.365
**Frequency of participants evaluated by Housing**	1147/266	0.996	0.987 - 1.006	0.439
**Exposure of sampling in 2011**	1158/269	1.214	0.726 - 2.032	0.459
**Sewer system**<br>Without connection to public network	1132/263	1.012	0.334 – 3.065	0.983
**Piped water**<br>Public network inside the house<br>Other	1132/263	Ref.<br>0.436	0.214 – 0.885	0.022
**Sanitary**<br>Direct discharge of water<br>Others	1132/263	Ref<br>1.331	0.758 - 2.338	0.319
**Flooring material**<br>Cement<br>Others	1132/263	Ref<br>2.118	0.650 - 6.899	0.213
**Number of rooms**	1132/263	1.018	0.901 - 1.150	0.772
**Inhabitants per room**	1127/262	1.024	0.780 - 1.344	0.864
**TV**	1132/263	1.091	0.126 - 9.473	0.937
**Refrigerator**	1132/263	4.843	0.645 – 36.356	0.125
**Washing machine**	1132/263	1.577	0.784 - 3.170	0.201
**Computer**	1125/261	0.488	0.251 – 0.948	0.034
**Poor housing maintenance***	1124/263	2.940	1.024 – 8.441	0.045
**Housing infestation****	1128/263	1.490	0.822 - 2.703	0.189

*Poor housing maintenance: houses were those having a poor structure, poor organization, dirty, with unpainted or cracked walls, improvised sections, or broken windows/doors
**House with larvae or pupae of *Aedes*.
