# Supplementary material for: Microclimate factors related to dengue virus burden clusters in two endemic towns of Mexico
Source: PLoS One. 2024 Jun 6;19(6):e0302025. doi: 10.1371/journal.pone.0302025 (PMC11156286; doi:10.1371/journal.pone.0302025)
Supplement: Attachment — Submitted filename: RESPONSE TO COMMENTS PONE-D-23-34212.pdf [file pone.0302025.s022.pdf]

PONE-D-23-34212
Microclimate factors related to dengue virus burden clusters in two endemic towns of Mexico

PLOS ONE
Dear colleagues,

The authors of this manuscript are thankful for the speed with which the review was carried out. In addition, we are grateful for the comments that have been made, which enrich the manuscript, and we provide a detailed answer to these comments below.

Journal requirements:

1. Please ensure that your manuscript meets PLOS ONE's style requirements, including those for file naming.

RESPONSE: We revised the manuscript and adjusted the text to the parameters required by Plos One. See text corrections in track changes.

2. Thank you for stating the following in the Competing Interests section:

Please confirm that this does not alter your adherence to all PLOS ONE policies on sharing data and materials, by including the following statement: "This does not alter our adherence to PLOS ONE policies on sharing data and materials."

Please include your updated Competing Interests statement in your cover letter; we will change the online submission form on your behalf.

RESPONSE: We confirmed that the Competing Interests statement does not alter the adherence to PLOS One policy, and therefore the suggested sentence was added to the corresponding section and additionally added to the cover letter.

3. In your Data Availability statement, you have not specified where the minimal data set underlying the results described in your manuscript can be found. PLOS defines a study's minimal data set as the underlying data used to reach the conclusions drawn in the manuscript and any additional data required to replicate the reported study findings in their entirety. All PLOS journals require that the minimal data set be made fully available. For more information about our data policy, please see http://journals.plos.org/plosone/s/data-availability.

RESPONSE: The authors of this study understood that the minimum data requested are intended to replicate the results independently; however, as the minimum data in this study refers to the address of the participants, the coordinates of the dwellings are protected by the federal law on the protection of personal data held by private individuals (https://iapp.org/media/pdf/knowledge_center/Mexico_Federal_Data_Protection_Act_July2010.pdf). Since the coordinates cannot be published on an open-access site,

queries for these data should be directed to the Chair of the Research Ethics Committee (https://www.insp.mx/insp-cei.html; comunicacion@insp.mx).

The data to develop the maps can be obtained from https://glovis.usgs.gov/app.

Accordingly, we added a section in the manuscript called Data Sharing with the following text:

The database with information from participants is unavailable since this project is covert by the Mexican federal law on protection of personal data held by private parties (information about the law can be found in: https:// privacyassociation.org/media/pdf/knowledge_ center/Mexico_Federal_Data_Protection_Act_ July2010.pdf). For specific inquiries about the data, interested subjects can contact the Chair of the Institutional Ethics Board (https://www.insp.mx/insp-cei.html; comunicacion@insp.mx).

The dataset of climate variables was constructed as described in the material and methods section; the maps with the information necessary to generate the variables can be obtained from https://glovis.usgs.gov/app **.**

4. We note that Figures 1,2,3,S1,S3,S4,S5,S6,S7,S8,S9,S10,S11 and S12 in your submission contain [map/satellite] images which may be copyrighted. All PLOS content is published under the Creative Commons Attribution License (CC BY 4.0), which means that the manuscript, images, and Supporting Information files will be freely available online, and any third party is permitted to access, download, copy, distribute, and use these materials in any way, even commercially, with proper attribution. For these reasons, we cannot publish previously copyrighted maps or satellite images created using proprietary data, such as Google software (Google Maps, Street View, and Earth). For more information, see our copyright guidelines: http://journals.plos.org/plosone/s/licenses-and-copyright.

We require you to either (1) present written permission from the copyright holder to publish these figures specifically under the CC BY 4.0 license, or (2) remove the figures from your submission:

RESPONSE. The authors of this study used the Esri module of ArcGIS. This module allows the access and publication of images from its libraries as long as at least one layer of information is added. We added the DENV infection and the climate information to the images. In the captions, we added the following credit as suggested by Esri: "Sources: Esri, DigitalGlobe, GeoEye, Earthstar Geographics, CNES/Airbus DS, USDA, USGS, AeroGRID, IGN, and the GIS User Community". We attach the current license, for using Esri-ArchGIS software, at the end of the response document and attach the Master Agreement for reference at this PDF.

Reviewers' comments:

Comments to the Author

Reviewer #1

The study is really interesting but some limitations are present.

1. How did the authors select two cities ? Please describe the criteria for choosing those selected two study areas in this study at the revised manuscript.

RESPONSE: We thank the reviewer for the comment. We accepted the suggestions and modify the paragraph in the section materials and methods. The new paragraph says: "Two dengue endemic towns in the state of Morelos, Mexico was selected taking into account the epidemiological information available up to the time of the study, which was provided by the Ministry of Health (SSA) of the federal government, where it was established that Morelos was the state with the most confirmed cases of dengue in Mexico. The first of them, Axochiapan (the urban area of the municipality of the same name) has approximately 18,659 inhabitants according to the 2015 INEGI estimated population; the temperature ranges between 13 °C and 35 °C. The second one, Tepalcingo (the urban area of the municipality of the same name) has approximately 12,895 inhabitants; the temperature varies between 19 °C and 34 °C (S1 Fig) [17-19].
Sources of information: A secondary analysis was conducted on data collected in a prospective population-based cohort study of people aged five years and older living in Tepalcingo and Axochiapan between 2014 and 2016. This cohort was assembled in 2011 and the objective of the primary study was to determine that dengue transmission occurs primarily in the peridomestic area of Index Cases (IC) in two Mexican endemic towns [5], which were selected because they were the localities in Morelos with rates of particularly high incidence of dengue, with similar population density and where at that time the vector surveillance program was being carried out by the Ministry of Health since 2008."

2. Panbio ELISA is the gold standard test but PBU 9-11 was equivocal result. How did the authors categorize for the equivocal results samples? Please describe clearly in the methodology section.

RESPONSE: We included Table 1 in the main text to explain the definitions of infection according to the stages of the study. In general, indeterminates were considered negative using all three techniques. In addition, the proportion of indeterminate results for each test was calculated to estimate their impact on the number of infections.

3.Anti-DENV IgM Ab can use for determining recent DENV infection. Please add the operational definitions for defining recent DENV infection in this study at the revised manuscript. What is the purpose for anti-DENV IgG ELISA? Please discuss and describe the justification for doing this one.

RESPONSE: In this sense, as mentioned in the previous point, Table 1 was added (please see the previous answer). On the other hand, for the capture of IgG, the Mexican algorithm for diagnosing Zika virus transmission considers the capture of

IgG as confirmation of dengue infection since, in Mexico, there was no other flavivirus circulating until November 2015 when Zika virus started to circulate.

4.DENV is one of the members of flaviviruses and how about the cross reactivity with other flaviviruses if the authors use only ELISA test for determining DENV infection. there is cross reactivity with other flaviviruses. We did not see any information about the cross reactivity of the tests for determining the recent DENV infection. Please briefly discuss about the limitation of the test used in this study.

RESPONSE: As mentioned in the previous answer, in Mexico and specifically in the study area, only the Zika virus started circulating in November 2015. As can be seen in Table 1, in surveys 3, 4 and 5, the result of IgG capture was excluded from the definition of recent infection, as the result of this test is particularly influenced by the cross-reactivity of flaviviruses; only those positive for IgM were considered DENV infections, as this test has a specificity greater than 90% using any diagnostic kit; the other criterion was seroconversion as measured by the previous paired sample in an indirect IgG assay that measures DENV exposure in historical samples; in this case, a negative result in the previous sample together with a positive result, even in the absence of positive IgM, is evidence of recent infection.
This is explained in the text as with Table 1 of the manuscript.

5.Why did not the authors choose NS-1 Ag tests which is available in RDT kit? If NS-1 Ag is positive, we can say very strongly that DENV infection is active and transmitted. Please discuss briefly on this issue.

RESPONSE: We disagree with the reviewer as this study aimed to detect total infections, not only symptomatic infections, which is the indication for the use of NS1 determination.

6. The authors described that many hotspots were identified in the study. But the authors choose IgM Ab for determing recent DENV infection. The IgM Ab can persists for three months. How can the authors say that the infected persons within hotspot got infection at the same period? Eg, In a hotspot one case got infection last three months ago and one case got infection at the time of travelling last one month. Although those houses of two cases were very close, we could not say this is active transmission of DENV at that hotspot. Only PCR can say and we can say same serotype or not/ Please also discuss on this issue.This is the disadvantages of using Ab only in this study. Please add as limitations of the study at the revised manuscript.

RESPONSE: We respectfully disagree with the reviewer. As explained in the previous answer, the project aimed to determine total infections, so we clearly state that we are studying recent infections, not active infections, as the reviewer suggests. This nuance is relevant because we intend to study transmission dynamics in the context of local climatic conditions, not disease incidence. Furthermore, we have evidence in these localities the dengue transmission mainly is peri domiciliary (Martínez-Vega RA, et al. PLoS Negl Trop Dis. 2015 Dec 15;9(12):e0004296.), considering that mobility outside

the community is not significant (Falcón-Lezama JA, et al. PLoS One. 2017 Feb 22;12(2):e0172313.)

Reviewer #2

The work by Tapias-Rivera and colleagues describes the analysis de microclimate factors related to dengue virus burden clusters in two endemic towns of Mexico. For the analysis they used collected data from previous studies performed during 2014 to 2016. The authors aimed to identify if dengue burden clusters are associated or related with two climate variables, LST and soil humidity.

The work is interesting and brings to discussion the use of clusters and hotspots as a methodological strategy to inform health authorities on high transmission zones.

However, there´s some concerns about the data presented by the authors.

1. There´s not clarity of the period of the study until the methods section.

RESPONSE: We thank the reviewer for the comment. We clarified the paragraph in the Introduction section to include the study period where the data were collected. The new phrase says: "Therefore, the study aimed to assess the association between some microenvironmental determinants and the occurrence of burden clusters of DENV infection in a population-based cohort in two endemic towns in southern Mexico from 2014 to 2016".

It should be clarified that data analysis is retrospective and not a "prospective population-based cohort study" as they mention on page 6 line 102.

RESPONSE: We are sorry, but we do not agree with the reviewer. The study group considers that the work corresponds to a prospective cohort study because the information collection process was prospective, including the consent process and the blood draw. However, the present manuscript reports the results of a secondary analysis, as this was not a specific objective of the primary project.

Thus, when authors mention "recent DENV infections" (although they define it) those are not related to current or new data. The word recent should be removed from all sections or tables or figures, it causes confusion. The study uses data collected from a decade ago.

RESPONSE: We understand the reviewer's comment, however, when the recent infection is mentioned, it refers to the fact that those were recent infections when the samples were processed for the study period analyzed. We don´t want confusion with a seroprevalence study therefore we use "recent infection" for clarity.

2. There´s not clarity on the data use and analysis when refer to clusters and hotspots. According to the source of information section, data are divided in those from people >5 years old collected during dengue transmission and those from the seroconversion dynamics study. But the analysis was performed with a section of the data, from participants who were assessed in five occasions. Then, its not clear why the analysis for each town is different. In the hotspots they described for participants from Axiochapan, results for surveys 1 and 3 but not mention what happen in the remaining surveys. However, for Tepalcingo, they described results for all five surveys. A similar description is found on the Microclimate analysis. If the data to perform the analysis its not significant for all five surveys for each town, it should be mentioned.

RESPONSE: We thank the reviewer for the comment, and we modified what was suggested in order to clarify the evaluation process during the 2.5 years of survey was the same for both localities; the new paragraph says:
"In Axochiapan, in surveys 1, 3, and 5 (August-November 2014, 2015, and 2016), 32 hotspots of recent DENV infection were observed predominantly in the southeastern area of the town. In Axochiapan surveys 2 and 4 (February-May 2015 and 2016), 14 hotspots were observed. However, autocorrelation analysis only identified significant clusters of recent DENV infection in six polygons in three neighborhoods in survey 1 (Vista Hermosa to the northwest and El Carmen and El Progreso to the southeast, with infection frequencies between 10% and 100%) and four polygons of two neighborhoods in survey 3 (El Carmen and El Progreso with infection frequencies between 5·6% and 33·3%) (Figs 1 and 2, S4 Table). The hotspots identified in surveys 2, 4, and 5 in this town did not consolidate as clusters of the burden of DENV infection."
Also, we add S3-S5 representing Axochiapan survey 2, 4 and 5 on the supplementary information file.

3. The numbers presented in S2 Figure are the totals for both towns. But the authors did not mention the numbers in each town. How significant is to perform the hotspots and microclimate analysis with a small number of cases, with 88 the highest and 8 the lowest in each survey? This should be mentioned in the results section and discussed.

RESPONSE: Figure S2 was modified to show the number of infections in each location. Furthermore, as the reviewer mentions, the number of infections is small, but as we find statistically significant results with this number of infections, the effect of climatic variables on dengue transmission should be even more significant than this study suggests. Therefore, we modified the Discussion section and added the following text to the limitations paragraph:
"Another possible limitation is that we were able to detect 8 infections in survey 4; However, there is a small number of infections; It was enough to determine a cluster with neighboring polygons in Tepalcingo"

4. Data discussed in page 16, lines 314 to 318 are overinterpreted. The author's comparison is with data reported in 2003. How data that were collected a decade ago by the authors can be applied during the actual dengue epidemiological surveillance system. How factors such

as population mobility, dengue seroprevalence, dengue outbreaks since 2014-2016 could affect what happened ten years ago? Although the burden clusters repeated for two years (2014-2016), before making any assumption, they should be compared with the actual situation.

RESPONSE: First, we would like to point out that the paragraph mentioned by the reviewer does not refer to any specific moment in time, so we do not know what the reviewer means by compare data from 2003. The reviewer mentions that the results are overinterpreted as the retrospective information does not allow comparison with the current situation, however, this report intends to describe how environmental variables influence the peridomiciliary transmission of DENV and thus eventually improve surveillance systems that currently, at best, only consider incidence and entomological data. In other words, this study does not intend to compare the dengue situation in these localities in 2003 with the current situation, so we disagree with the reviewer's comment.

Reviewer #3

1.Definition of Recent DENV infection is not clear. The author want to say DENV IgM positive or DENV IgM and IgG positive?Please calrify it.

RESPONSE: We appreciate the suggestion and revised and included Table 1 in the main text to explain the definitions of infection according to the stages of the study.

2. The author did 5 surveys. Survey 1 showed the highest recent DENV infection. Why? Survey 1 period (Aug-Nov 2014) is big dengue outbreak in that area or other reasons?

RESPONSE: The study group considers this situation because, according to the Epidemiological Panorama of dengue, in 2014, there were more cases in Morelos than in the following two years. In particular, a locality (Tepalcingo) was reported in the state's second-highest number of cases. (https://www.gob.mx/salud/documentos/direction-general-de-epidemiologia-panorama-epidemiologico-de-dengue -2014-semana-epidemiologica-53; https://www.gob.mx/salud/documentos/direction-general-de-epidemiologia-panorama-epidemiologico-de-dengue-2016-semana-epidemiologica-52)

Accordingly, we add the following text: *“Moreover, in 2014, more dengue cases were reported by the surveillance system in Morelos than in the following two years. In particular, Tepalcingo had the state's second-highest number of cases.”*

3. Based on Survey 1-5, 2014 the highest % recent infection, 2015 lowest % recent infection and 2016 recent infection higher than 2015, but lower than 2014. It is better to discuss recent infection trend condition in the discussion.

We thank you and accept the recommendation. In the discussion section, we include a text about this. The new sentence says: "with the known cyclical incidence rate

described in many studies in endemic cities" (Wearing HJ, Rohani P. Ecological and immunological determinants of dengue epidemics Proc Natl Acad Sci U S A. 2006 ;103(31):11802-7 and Adams B, Holmes EC, Zhang C, Mammen MP, Jr., Nimmannitya S, Kalayanarooj S, et al. alternating epidemic pattern of dengue virus serotypes circulating in Bangkok, Proc Natl Acad Sci U S A. 2006;103(38):14234-9).

4. land surface temperature, soil humidity were not independently associated with DENV burden clusters. So, which possible factors are associated with DENV burden? please discuss it.

RESPONSE: We are confused. As mentioned in the results section, "Hexagonal LST was negatively associated with households belonging to a DENV loading group, while high soil moisture had a positive association with this outcome (Table 2). Therefore, that is exactly the observation, that climatic factors determine the burden clusters.

5.Significant clusters of recent DENV infection described in Axochiapan (S4) and Tepalcingo (S5). Recent infection % are different on Survey period. But it is clearly to explain in result part.

RESPONSE: We regret that we do not understand what the reviewer is commenting on and therefore cannot give a detailed answer. However, the response to reviewer 2's comment 2 may be associated with this observation.

6. Supplementary data are too much. It is better to omit some figures and tables.

RESPONSE: We appreciate the reviewer's recommendation, however, in the opinion of all the authors, the supplementary information is the minimum sufficient to understand the entire manuscript. Therefore, no further editing was done in this regard.

Ciudad de México, 24 noviembre 2023

Cliente	Usuario
**Instituto Nacional de Salud Pública**<br>Subdirección de Apoyo Académico con funciones de Geografía Médica y Geomática	**René Santos Luna**<br>rsantos@insp.mx

Por este medio Sistemas de Información Geográfica, S.A. DE C.V. manifiesta que en cumplimiento a su Orden de compra que incluye:

DESCRIPCIÓN	Cantidad	Años
Licencia Departamental Pequeña (5 usuarios).	1	1

Los entregables se realizan de acuerdo con lo siguiente:

1. **Software de escritorio ArcGIS Pro**

Medios de Instalación (acceso a página Web My Esri)

ArcGIS Pro y otros componentes están disponibles para descarga a través del sitio My Esri.
Nuevos usuarios: para tener acceso se debe crear una cuenta siguiendo el manual que se comparte en el email de entrega de licenciamiento. El acceso a este sitio estará habilitado hasta el **23/11/2024**. La persona que tiene habilitado el acceso a este sitio es: **René Santos Luna** con la cuenta de correo electrónico: rsantos@insp.mx
Este sitio se actualiza de forma automática e inmediata cada vez que se libera una nueva versión del software. Se recomienda hacer una revisión periódica.

- Solicitudes de casos de soporte a: soporte_esri@sigsa.com.mx o en nuestro sitio de soporte técnico SIGSA|ESRI.

Medios de Instalación (acceso a página Web ArcGIS Online)

Si se trata de **nuevo usuario** deberá activar su cuenta de organización (proporcione una fecha de reunión disponible para ello a: carolina.teutle@sigsa.info) Una vez dentro de ArcGIS Online, puede descargar ArcGIS Pro a través de su cuenta activa, siguiendo los pasos de descarga e instalación.
Si se trata de renovación puede acceder a ArcGIS Online con su usuario y contraseña y seguir los mismos pasos de descarga e instalación.
Más recursos sobre instalación de ArcGIS Pro:

- Video

IMPORTANTE: Antes de instalar ArcGIS Pro 3.0.x se requiere instalar Microsoft .NET Desktop Runtime 6.0.5 o una versión de parche posterior (6.0.6 y así sucesivamente), con un instalador de Windows x64. La presencia de Microsoft .NET Desktop Runtime 7 o posterior es intrascendente.

2. Licencia

Los códigos de activación para su **Licencia Departamental Pequeña (5 usuarios)** son los siguientes:

ArcMap---

EFL265560XXX	ArcGIS for Desktop Advanced
EFL710018XXX	ArcGIS Geostatistical Analyst for DTOP
EFL1839079XX	ArcGIS Data Interoperability for Desktop
EFL4075615XX	ArcGIS Workflow Manager for Desktop
EFL3155856XX	ArcGIS LocateXT for ArcGIS Desktop
EFL5154360XX	ArcGIS Network Analyst for Desktop
EFL541071XXX	ArcGIS Publisher for Desktop
EFL887989XXXX	ArcGIS Data Reviewer for Desktop
EFL030867XXX	ArcGIS 3D Analyst for Desktop
EFL499255XXX	ArcGIS Tracking Analyst for Desktop
EFL110757XXX	ArcGIS Schematics for Desktop
EFL3666066XX	ArcGIS Spatial Analyst for Desktop
EFL1489488XX	Esri CityEngine Advanced

IMPORTANTE: Este es el ultimo año que se hará entrega de licenciamiento ArcMap. Favor de iniciar un plan para migrar a ArcGIS Pro. Recursos en: Migrar a ArcGIS Pro

ArcGIS Pro--

EFL382788XXX	ArcGIS Pro Advanced
EFL1068973XX	ArcGIS Pro 3D Analyst
EFL7607597XX	ArcGIS Pro Data Reviewer
EFL6943422XX	ArcGIS Pro Data Interoperability
EFL540218XXX	ArcGIS Pro Geostatistical Analyst
EFL331329XXX	Image Analyst for Pro
EFL468031XXX	ArcGIS Pro Network Analyst
EFL8369655XX	Publisher for Pro
EFL189286XXX	ArcGIS Pro Spatial Analyst
EFL191145XXX	ArcGIS Pro Workflow Manager
EFL939006XXX	ArcGIS LocateXT for ArcGIS Pro

IMPORTANTE: Este es el último año que se hará entrega de códigos para uso concurrente. Favor de revisar la asignación a través de Named User.

Enterprise---

ECP937707XXX	ArcGIS GIS Server Advanced
ECP2884743XX	ArcGIS Data Reviewer Server Extension
ECP036459XXX	ArcGIS Workflow Manager Server Standard
ECP8274466XX	ArcGIS Workflow Manager for Server
ECP494840XXX	Data Interoperability Server
ECP9253100XX	ArcGIS Notebooks Standard

El periodo de mantenimiento vencerá el 23/11/2024

3. Acceso a ArcGIS Online

El periodo de vigencia vence el **23/11/2024**. La información correspondiente es la siguiente:

ArcGIS Online
Subscription ID: 3974976499
Self-Help Resources<br>ArcGIS Online help<br>ArcGIS Online forum<br>ArcGIS Online videos<br>ArcGIS Online blog

4. Soporte técnico

Soporte para la activación de cuentas My Esri, ArcGIS Online **y licencias:** Este lo estará proporcionando Maura Pérez, con cuenta de correo seguimiento_esri@sigsa.info.
En el sistema de soporte técnico, nosotros debemos dar de alta a un contacto, quien será el responsable de levantar los casos, y es a quien soporte estará respondiente y/o estableciendo contacto. La persona que se está dando de alta en soporte Técnico es: **René Santos Luna** con la cuenta de correo electrónico: rsantos@insp.mx Reportar el caso a través del sitio My Esri. My Esri tiene un apartado para Soporte. El sistema genera un ticket que recibe el equipo de Soporte técnico.

- Tienen también la opción de enviar correo electrónico a seguimiento_esri@sigsa.info
- En el reporte deberá describir de forma sencilla el problema indicando nivel de licencia y versión, así como su nombre, y cuenta de correo.
- El sistema al recibir el reporte de soporte mandara una confirmación de recepción y le asignara un número de caso. La respuesta incluye ya el número de caso.
- El servicio de soporte técnico se proporciona en idioma español de lunes a viernes, con un horario de atención de 9:00 a 19:00 horas.
- El soporte técnico estará habilitado hasta el 23/11/2024
- Correo electrónico de atención: soporte_esri@sigsa.com.mx
- EL nombre de la(s) persona(s) encargada(s) de brindar el soporte: lo asigna el sistema en el momento de recibir el reporte de soporte
- Número de reporte o ticket de incidencia: lo asigna el sistema en el momento de recibir el reporte de soporte

Entrega

Ejecutiva de ventas | Educación
Carolina Teutle Ixehuatl
Sistemas de Información Geográfica, S.A. de CV

Recibe

René Santos Luna
