# Supplementary material for: Microclimate factors related to dengue virus burden clusters in two endemic towns of Mexico
Source: PLoS One. 2024 Jun 6;19(6):e0302025. doi: 10.1371/journal.pone.0302025 (PMC11156286; doi:10.1371/journal.pone.0302025)
Supplement: Attachment — Submitted filename: Response to 2nd revision.docx [file pone.0302025.s023.docx]

**Response to PONE-D-23-34212R1, Microclimate factors related to dengue virus burden clusters in two endemic towns of Mexico.**

On behalf of all the authors I would like to thank the editor and reviewers for their comments. Here we respond to the comments expressed by the reviewers in detail.

Reviewer #1: (No Response)

Reviewer #2: The authors have addressed some of the comments from previous revision. However, there´s still lack of clarity on the source of information section and recent dengue infection section.
It is suggested to the authors to re-write the Source of Information Section. According to what they described, a cohort was first assembled in 2011 (reference 5 from the same team). This cohort included 862 participants. Then, for the secondary study performed during 2014 to 2016, they added 120 participants; although this number doesn't matches the number (982) on the text (page 6 line 116) neither the number on figure S2 (966 participants).
A confusion also comes when they mention in page 6 lines 114-118 the following... This analysis included the subgroup of persons evaluated during the second phase of the cohort study who had participated during the first phase of the cohort and were DENV seronegative or had a DENV infection (n=461 of 862 included between August 2011 and March 2012)[5], or who were recruited in the second phase of the cohort and were DENV seronegative (n=19 of 120 included between August and November 2014) (S2 Fig).
Then, for the five serosurveys, they included different participants from the total of 966? or 982? or 862? Clarify which is the total number of participants in this study.

**RESPONSE**: We are grateful to the reviewer for bringing this typographical error to our attention and it has been corrected as mentioned below (changes in bold):

Line 120, page 7: "...DENV seronegative (n=19 of **104** included...).

We also corrected a confusing paragraph as follows:

Line 116-117, page 6: "...had a **recent** DENV infection, **or were randomly selected from seropositive participants without DENV recent infection**..."

We hope that with these changes the point made by the reviewer is clarified.

The section Recent Dengue Infection. The authors explained the reasoning to consider "recent infection" although it was suggested to eliminate this word. They argue the fact that only Dengue was in circulation before Zika introduction. What would be the result after Zika introduction in 2015? they did survey 3 in the second semester of 2015 and surveys 4 and 5 in 2016 when even Chikungunya was introduced in Mexico.

**RESPONSE:** We disagree with the reviewer about removing the definition of recent infection, for two reasons.

1) The reviewer, without explicitly mentioning it, suggests that due to antigenic cross-reactivity with other flaviviruses a serological definition of recent infection cannot be used. However, if there is no other flavivirus circulating in the area, how could IgM against DENV be positive, or, for that matter, what infection would cause IgG seroconversion in the indirect IgG testing for DENV? The reviewer mentions the case of Chikungunya virus, which has certainly circulated in Mexico since 2014, although transmission in the study state was reported only in 2015 (https://www.gob.mx/salud/acciones-y-programas/historico-boletin-epidemiologico); in any case, Chikunganya virus is not antigenically related to Dengue virus, because it is an alphavirus of the *Togaviridae* family. Thus, it was only until the report of Zika virus circulation in Mexico in 2015 that it was decided to omit the IgG capture test from the definition of recent infection, because of the possibility of antigenic cross-reaction, but we should mention that only until 2016 was Zika virus circulation reported in Morelos (op cit).

2) In at least three previous publications, Martínez-Vega RA, et al. PLoS Negl Trop Dis. 2015;9(12):e0004296; Falcón-Lezama JA, et al. PLoS One. 2017;12(2):e0172313 and Amaya-Larios IY, et al. Sci Rep. 2020;10(1):19017, we used an identical or very similar definition when describing cases of recent DENV infection in the cohort.

Therefore, we stand by our definition of recent infection as described throughout the manuscript.

The selection of the two sites for performing the study was according to the authors, because were dengue-endemic localities in Morelos with high incidence rates. However, the authors never mention in the document which was this incidence, nor the number of dengue cases reported by the Health Ministry.

They mention in the Discussion section (page 17, lines 311-312).... Moreover, in 2014, more dengue cases were reported by the surveillance system in Morelos than in the following two years. In particular, Tepalcingo had the state's second-highest number of dengue cases. Include the numbers.

The authors can revise the following article: https://www.medigraphic.com/pdfs/infectologia/lip-2020/lip202d.pdf. In page 80, there´s a summary of confirmed cases of Dengue from 2000 to 2019.

**RESPONSE**: The reference suggested by the reviewer mentions cumulative dengue cases in Mexico, which does not contribute to resolve the point the reviewer mentions; however, to meet the reviewer's observation we add a reference, Martínez-Vega RA, Danis-Lozano R, Velasco-Hernández J, Díaz-Quijano FA, González-Fernández M, Santos R, Román S, Argáez-Sosa J, Nakamura M, Ramos-Castañeda J. A prospective cohort study to evaluate peridomestic infection as a determinant of dengue transmission: protocol. BMC Public Health. 2012 Apr 2;12:262, which details the epidemiological indicators requested by the reviewer, specifically for the two studied localities.

Therefore, the Study area of the Methodology section was modified with the following text: **Details of dengue transmission in the selected localities can be found in Martinez-Vega RA, et al BMC Public Health. 2012;12:262.**

Reviewer #3: The revised manuscript addressed my comments. there has no more comments for me. I accepted revised manuscript.

José Ramos-Castañeda PhD
